# Influence of Socio-Demographic Factors in the Promotion of Social Entrepreneurship: A Service-Learning Programme

**DOI:** 10.3390/ijerph182111318

**Published:** 2021-10-28

**Authors:** María Maravé-Vivas, Celina Salvador-Garcia, Carlos Capella-Peris, Jesús Gil-Gómez

**Affiliations:** 1 Department of Pedagogy and Didactics of Social Sciences, Language and Literature, Universitat Jaume I, 12071 Castellón, Spain; marave@uji.es; 2 Facultad de Educación, Universidad Internacional de la Rioja, 26006 Logroño, Spain; salvadoc@uji.es; 3 Physical and Sports Education Department, Universitat de València, 46010 València, Spain; 4 Department of Education and Didactics of Specific Subjects, Universitat Jaume I, 12071 Castellón, Spain; jegil@uji.es

**Keywords:** service-learning, higher education, social entrepreneurship, physical education, active methodology

## Abstract

Social entrepreneurship (SE) is often presented in the literature as the key to solve many of this world’s persistent social problems. SE offers a special opportunity to address the 2030 Agenda for Sustainable Development and to boost the Sustainable Development Goals (SDGs). This research examines the effects of Service Learning (SL) on the SE of university students and to examine whether certain sociodemographic factors (i.e., age, entrance studies, family studies, social participation, and employment situation) are associated with students’ SE competence development when applying SL. Pre-service teachers (n = 98) of the degree in early childhood education applied a physical education SL programme. We used a quantitative method with a pre-experimental design, using pre-test and post-test measures. The findings obtained show a significant improvement on the SE competence of PSTs, so SL seems a good tool to develop it. The results that analyse the influence of socio-demographic factors do not show significant correlations. There are very few studies focusing on this objective, so it would be interesting to encourage the research community to provide more data in this area.

## 1. Introduction

In recent decades, higher education has been shifting from a directive and instructional approach to the use of active and experiential methodologies. This situation allows students to apply the acquired competences in real contexts, which is of vital importance in teacher education. In this vein, service learning (SL), is a methodology based on John Dewey’s experiential learning theory [1]. SL seeks to develop academic competencies and increases reflection while providing a community service to meet social needs. In other words, when applying SL programmes, participant students engage in activities aimed at addressing human and community needs, and this experience leads them to reflect on their practice and gain further understanding of the course content [2]. The implementation of SL shows improvements in personal, social, and professional dimensions, promoting social skills, improving emotional engagement and cognitive readiness with the community, and increasing evaluation skills in pre-service teachers (PSTs) [3,4,5], who are university students attending teacher education courses. Therefore, SL stands as an optimal strategy to increase personal, professional, and social wealth in students and society, through a comprehensive implementation, merging theory, and practice [6]. In addition, SL has promoted innovative and entrepreneurial learning experiences [7], which are critical in our research.

A surge of research interest in social entrepreneurship (SE) has flourished in the last decade, providing important insights regarding its role in fostering inclusive growth and institutional change [8]. Although there are some discrepancies regarding the conception of SE among scholars and practitioners [9,10,11,12], it is accepted that the main objective of SE is to increase social value in the community [13], which is in clear contrast to other entrepreneurial approaches, such as commercial entrepreneurship. Indeed, SE is often presented in the literature as the key to solve many of the world’s persistent social problems [11]. Therefore, our approach to what SE consists of follows the ideas of Capella et al. [12], who consider that SE is a competency leading towards construction, evaluation, and pursuit of opportunities for social change combating group disadvantage. In addition, it should embrace both social and entrepreneurial aspects. For this reason, we believe that SE offers a special opportunity to address the 2030 Agenda for Sustainable Development and to boost its 17 sustainable development goals (SDGs) (https://sdgs.un.org/goals accessed on 27 October 2021). Specifically, our proposal, which focuses on the promotion of SE in PSTs, may be useful to enhance goal 1: economic growth must be inclusive to provide sustainable jobs and promote equality (no poverty); goal 4: obtaining a quality education is the foundation to improving people’s lives and sustainable development (quality education); goal 5: gender equality is a necessary foundation for a prosperous and sustainable world (gender equality); goal 10: to reduce inequalities, policies should be universal in principle, paying attention to the needs of disadvantaged and marginalized populations (reduced inequalities); goal 16: access to justice for all, and building effective, accountable institutions at all levels (peace, justice, and strong institutions); goal 17: revitalize the global partnership for sustainable development (partnerships).

In this sense, different studies with trainee teachers suggest that SL can foster knowledge, understanding, sensitivity and commitment in relation to the SDGs [5,14]. It is, therefore, an appropriate methodological approach for acquiring professional skills and social values which are not only related to the SDGs [6] but also to SE [15]. In fact, according to Capella et al. [15], SL is an educational experience of great value at personal, social, and innovative levels, because it pursues social change combating social needs and disadvantages [12]. Bearing in mind the links between the effects of SL, and some SDGs and SE, there is a need to specifically examine whether applying this methodology can foster them in specific contexts.

Driven by previous literature requests, this research analyses the effects of SL in the field of physical education [15,16,17,18]. A number of works have examined the impact of this methodology specifically on personal, civic, or social outcomes [19,20]. However, our aim is to uncover the global effect on these areas, analysing the influence of SL on social entrepreneurship competence (SEC) in the teacher training context.

Previous research examining the effect of SL on SE in a North American university suggests that SL participation in the physical education field comes with an improvement in students’ social, personal, and innovative traits [15]. Therefore, in addition to assessing the effects that the application of a particular methodology has on university students in a Spanish context, the unique contribution of this investigation lies in the fact that it is important to analyse personal factors that may influence these results. In this sense, at university, we find students with very diverse socio-demographic factors that can condition the effects that a particular methodology produces in them. There is very little research on the influence of these factors on SL. Based on the limited literature on the subject, factors such as age, gender, previous participation in volunteering, previous studies, income, and ethnicity influence some aspects of students’ civic attitudes and skills [21,22,23,24,25,26].

We consider it interesting to investigate the influence of socio-demographic factors, as the results may provide us with clues or recommendations that can serve as a guide for the implementation of future teaching innovation projects or for teachers to choose a certain strategy/include it or assess it in relation to the students they have to attend to and their characteristics.

The present research has two objectives: to analyse the effects of SL on the SE of university students and to examine whether certain sociodemographic factors (i.e., age, entrance studies, family studies, social participation, and employment situation) are associated with students’ SEC development when applying SL. In this way, we will be able to compare the results obtained in each of the factors under study.

## 2. Hypotheses

Two hypotheses are put forward, on the one hand, that participation in the SL programme will significantly improve the SEC of university students, and on the other, that socio-demographic factors such as age, university entrance studies, family studies, previous participation in volunteering or associations, and employment situation will have an influence on the effects produced by the use of SL on the students’ SEC.

## 3. Materials and Methods

### 3.1. Design and Participants

This intervention experiment used a quantitative method with a pre-experimental design based on pre-test and post-test measures carried out with 98 PSTs. It examined the effect of participation in the SL programme on SEC and contrasted how sociodemographic factors may have been related to such an impact.

All participants attended the annual course subject “Fundamentals of body language; motor games in early childhood education”, present in the 2nd year of the degree in early childhood education at Jaume I University, during the 2017/2018 academic year. This subject is focused on the development of contents of the physical education field, which are essential to promote children’s integral development. In addition, due to the idiosyncrasy inherent in this subject, a number of enriching interactions are promoted between PSTs and the children receiving the service.

The sample was formed from a non-probability sampling, because it used a natural group and showed a bias regarding sex, because the majority of the students were female. This fact ruled out the gender variable for the investigation. However, such a distribution is not new, since most of the time, the number of females is higher in this context [27], a circumstance that has been corroborated by previous studies on SL in early childhood education [28,29].

### 3.2. Programme

**Figure 1:**
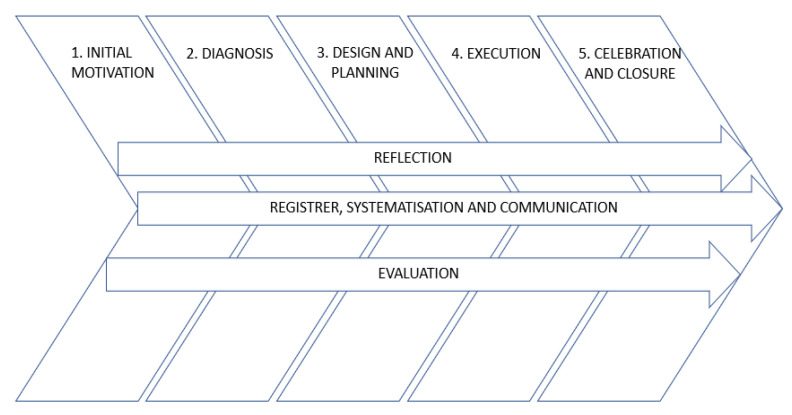
CLAYSS (2016) model phases.

According to the curriculum and course syllabus, the reference subject aims to develop students’ skills to work body language in early childhood as well as to develop their motor domain through motor games. SL methodology was a strategy applied to develop the competences that PSTs had to acquire. Therefore, an SL programme was embedded in the subject. The design of such a programme was based on the quality guidelines established by the National Youth Leadership Council [30]; in addition, the CLAYSS [31] model was applied. This model is divided into five phases (initial motivation, diagnosis, design and planning, execution, and lastly, celebration and closure) and three transverse processes (reflection; register, systematisation, and communication; and evaluation) Figure 1 shows this model.

Within this programme, students were divided in groups of four or five members to carry out a direct SL project aimed at children with functional diversity and/or suffering from social exclusion. These children had some kind of motor and expressive impairment as a shared feature. Each group designed and carried out their own project. It was sketched bearing in mind the motor and social needs of the children, aspects that PSTs had identified in the diagnosis phase. Thus, the PSTs project consisted of designing and carrying out practical body language and motor game sessions with the aim of improving these areas as well as promoting social inclusion. The students could choose among six local organizations that had agreed to be involved in the programme. Each organization had a particular schedule; consequently, the projects could vary in terms of total length. In any case, all the groups were engaged in approximately eight hours of direct service (on average, this meant three month projects of one or two weekly sessions). In addition to the time in direct contact with the children, PSTs had to undertake the rest of the phases integral to SL methodology. As a result, each pre-service teacher dedicated a total of 25 h on the project.

### 3.3. Instrument

A Spanish version of the *Social Entrepreneurship Competency Scale* (SECS) instrument was used [12]. This tool has 30 items that allow researchers to measure SE considering 3 categories of features (i.e., personal, social, and innovative) and 17 specific features (i.e., confidence, goal-oriented motivation, ability to take risks, ability to learn and evolve, creativity, offering help and cooperation, social awareness, coexistence and respect for public affairs, resilience, responsibility, commitment and coherence, ability to create ideas, leadership, initiative, ability to change, belonging to well-informed social networks, and ability to identify opportunities). Some item examples are as follows: “I believe I am capable of dealing with most situations” (for confidence); “People who help others are an example to follow” (for social awareness); and “I like coordinating other people while working in collaboration” (for leadership).

### 3.4. Data Analysis

Table 1 presents the socio-demographic factors examined in this research together with the groupings made and the percentages within the sample.

Data were analysed using SPSS Version 26, using descriptive and inferential statistics. Specifically, *t*-tests for comparison of means and analysis of variance models (ANOVA) were used.

## 4. Results

In order to examine the first hypothesis of the research, a reliability test was first carried out. A value of *α* = 0.896 was obtained for Cronbach’s Alpha test, showing good internal consistency [32]. Then, pre-test/post-test comparisons were made to the test using the paired samples *t*-test. The results showed significant differences (*p* < 0.05) for the SECS overall, as well as for all feature categories (Table 2).

In addition, the analysis by individual features showed significant differences (*p* < 0.05) for the ability to take risks and the ability to learn and evolve. The results for these features after applying the paired samples *t*-test were *t*(2) = −6.928, *p* = 0.020 and *t*(1) = −35.000, *p* = 0.018, respectively. However, we should note that 15 of the 17 features that make up the SEC improved in value from the pre- to the post-measurements. Creativity showed the same result for both measurements and only the ability to identify opportunities scored lower for the pre-test compared to the post-test measurement (Table 3).

The second hypothesis establishes that socio-demographic factors such as age, university entrance studies, family studies, previous participation in volunteering or associations, and employment situation will have an influence on the effects produced by the use of SL on the students’ SEC. Bearing in mind the fact that this competence has improved significantly, an analysis of variance (ANOVA) with repeated measures was carried out to examine the individual effect of the seven factors on the results of the SECS between pre- and post-test measures. To do so, each of the factors was entered as a covariable in the ANOVA. Table 4 displays the results of this analysis. 

Results show significant differences when both analyses are combined and the global pre-test/post-test results of the SECS are compared. However, when using the ANOVA to analyse the influence of the factors with repeated measures, these do not appear for any of the six factors (*p* > 0.05). This means that all the factors influence the results of the scale.

Given that the study factors are grouped with different structure (see Table 1), it is convenient to analyse the SECS results considering each factor separately. Thus, ANOVA tests were performed on post-test scores to analyse significative changes. Table 5 lists the results for each factor.

No significant differences were obtained in the analysis by factors. However, it is interesting to highlight some outcomes that arise from the analysis of the mean scores, which we summarize as follows: better mean scores are found as age increases; students who accessed from the high school achieved better social entrepreneurship results; the higher the family education, the better the SECS scores; having previous experience on social participation favoured the development of social entrepreneurship; participants who considered themselves progressive or liberal obtained better scores in social entrepreneurship after using SL, although the difference is small compared to those who defined themselves as conservative; and having work experience enhanced the acquisition of the social entrepreneurship features as a result of applying SL.

## 5. Discussion

The present study examines the impact of a SL programme applied through a subject within the field of the Didactics of Physical Education on the participant PSTs. Particularly, the first hypothesis focuses on the effects related to participants’ SEC, and the second on the sociodemographic outcomes that may influence such results.

The findings obtained show a significant improvement on the SEC of PSTs; thus, the first hypothesis is accepted. Previous research had examined the effects of experiential methodologies applied in different contexts on students’ SEC [33] as well as the integration of social responsibility in entrepreneurship education [34]. However, its analysis concerning SL programmes is still limited. For example, a theoretical paper on this issue was previously published [35], whereas other authors detailed a practical proposal [36]. Both texts assert that SL may emerge as an adequate methodology to develop students’ SEC. However, to our knowledge, only two studies have approached these ideas from an experimental lens. A study empirically analysed the impact of SL on participants’ SEC [15]. They implemented a mixed methods study, and both quantitative and qualitative approaches supported that there was an improvement in SEC outcomes, thus being in accordance with the results found in our study. On their part, other work conducted semi-structured interviews and analysed learning diaries to understand how SL participation had an impact in students’ SEC [37], and their results underlined that SL approaches (compared to traditional formats) had an increased impact on students’ competences. Nevertheless, their investigations were carried out in the North American context and in Germany, respectively, and involved a limited number of participants. This means that our study contributes new data supporting their findings in a different setting.

SEC, however, may be divided into different categories of features, namely personal, social, and innovative [12], and PSTs participating in the SL programme of the present study reported significant improvements on these three categories. The branch focused on personal features is linked to a reformulation of personal values and beliefs concerning the pre-service teachers’ way of relating to others [38], and its development has been previously reported by literature on SL in the physical education field [15,39]. Similarly, social skills have been widely uncovered when applying SL in the physical education field too, and the literature asserts that this methodology is an optimal way of addressing social transformation issues [20,40], because it enhances participants’ social sensitivity [41,42].

In fact, according to a recent systematic review on SL on physical education teacher education, many studies of the sample analysed focused on the impact of SL methodology on higher education students’ personal and social skills [18]. In addition, a different work designed a scale to assess physical education SL in higher education in order to measure students’ perception of participation in university SL experiences [43], and this instrument focuses on the contribution of this methodology to personal and social development besides of learning. These ideas support the relevance of the findings of our study, since they highlight the relevance of improving both personal and social categories of the SECS through SL in the field of physical education [27,44].

With regard to the third category of features, which is related to innovative skills, the literature is less developed. Although research on social innovation is one of the four strands in which entrepreneurial social value creation is divided [10], the literature on SL has scarcely examined the improvement of this skill on the part of students [15]. However, the enhancement of this ability is relevant in the teacher training field, since the development of innovative features may mean that PSTs will be more prone to participate in and design new and innovative projects in the near future, concerning not only their personal life, but also professional settings when they become qualified teachers.

The SECS has 17 specific features, among which 15 displayed an improvement in their value when pre-test and post-test scores were compared. This result is similar to that achieved by some authors [15], who found higher scores of 16 of these specific features when the post-test measures of experimental and control groups were compared. In our case, the differences found achieved significant levels only in the specific features concerning ability “to take risks” and “to learn and evolve”. Regarding the former, the literature ascertains that physical education PSTs’ confidence in their abilities to support diversity and to want to teach physical education was enhanced after participating in SL [44,45,46,47]. Thus, this increased confidence may have helped to improve participant PSTs’ ability to take risks in our study. Concerning the latter, SL seems to entail a turning point for participants in terms of evolution [48] and learning [19,20,27,40,49], because students tend to consider it a *life-changing* experience [48].

The second hypothesis argued that sociodemographic factors could influence the participants’ SEC, but according to the results obtained, it has to be rejected, since no significant effects have been found. This finding is not surprising, since literature on SL examining the influence of students’ sociodemographic features is inconclusive. For example, regarding the age factor, two studies found that this feature could have an effect, and both agree on the fact that the older the students, the more significant the results [23,24]. However, a different study examined students’ empathy, and it concluded that the older group of participants did not increase in this feature, whereas students younger than 25 did report significant improvements [17]. Other authors, on their part, found that students’ satisfaction with the SL programme was high in all cases, regardless of their age [50]. Therefore, the ways in which age affects the outcomes achieved after SL seem to be unclear and need further consideration.

The way of entering the university and relatives’ educational level could be other factors influencing the results of the present study, since they have been relevant in some cases regarding outcomes related to empathy and social and civic attitudes [17,25]. However, it has not been the case for SEC, and thus, research focusing on these topics from a qualitative perspective could be useful to gain understanding on this factor.

Moving now to the factor focused on previous social participation, the literature suggests that volunteering or similar activities with underserved communities may be influenced by the participants’ previous experiences [51]. In fact, a study considers that not taking into account students’ prior experiences working with socially disadvantaged people was a limitation of their investigation [52]. Furthermore, another research concluded that engaging in SL led to higher participation in general volunteering [53]. However, the present study did not find this type of influence, which is in line with a previous research [24].

Finally, employment status did not show any significant correlation with SEC development, similarly to previous results concerning social and civic attitudes [25]. However, this variable was considered to be relevant in other occasions, because, sometimes, when people are employed full time or even part time, they may encounter some difficulties, such as the incompatibility of schedules, in actively participating and making the most of the SL experience [50,54]. In any case, the results of the present study suggest that employment status was not a constraint for participants’ SEC improvement. 

Despite the interesting results obtained, there exist some limitations that should be taken into consideration. For example, there was not a control group, and students were selected by means of convenience sampling. In this sense, a randomized controlled trial would have strengthened the validity of this study [55]. In addition, the sample is not representative of any large population due to gender bias; therefore, findings cannot be generalized, and the validity of this research may have also been compromised [56]. However, the gender variable was not considered for this investigation, and this unequal distribution is frequent in this type of context [27], as previous studies on SL in early childhood education have stated [28,29].

## 6. Conclusions

This research has presented the results of applying a SL programme with PSTs in the field of physical education, aimed at analysing the effects of SL on the SEC of university students and examining the influence that socio-demographic factors may have on these effects. On the one hand, the results obtained show that the SL programme applied in the field of PSTs training through physical education is a useful tool for developing SEC. On the other hand, the analysis of the influence of socio-demographic factors does not show significant effects. There are very few studies focusing on this objective, so it would be interesting to encourage the research community to provide more data in this area. If research is carried out in this sense, it may offer clues for university teachers in the design and implementation of SL programmes in relation to the features of their students.

## Figures and Tables

**Table 1:** Factors examined, groups and percentages within the sample.

Factors	Groups					
Age	≤21		22–25		>25	
Percentage	46.9		46.9		6.1	
Education Access ^1^	HS		VC		HS&VC	
Percentage	57.1		43.7		7.1	
Family education	Other	Basic		Medium		Higher
Percentage	1	14.3		69.4		16.3
Previous social participation	Yes			No		
Percentage	44.9			55.1		
Employment status	Works			Does not work		
Percentage	37.8			62.2		

^1^ High school (HS), vocational training (VC), first high school, then vocational training (HS and VC). Data obtained in the research (own elaboration).

**Table 2:** Pre-test/post-test comparisons.

Paired Samples Test								
	Paired Differences							
	Mean	Standard Deviation	Deviation Error Mean	95% Confidence Interval of the Difference		*t*	df	Sig. (Bilateral)
				Lower	Upper			
Social entrepreneurship	−0.0846	0.077	0.014	−0.113	−0.056	−5.995	29	0.000
Personal features	−0.0844	0.066	0.022	−0.135	−0.034	−3.851	8	0.005
Social features	−0.0818	0.078	0.023	−0.134	−0.029	−3.475	10	0.006
Innovative features	−0.0880	0.093	0.029	−0.154	−0.021	−2.994	9	0.015

**Table 3:** Pre-test/post-test values by features.

Feature	*Mean (Standard Deviation)*	
	*Pre-Test*	*Post-Test*
Leadership	3.980 (0.056)	4.065 (0.078)
Goal-oriented motivation	3.700 (0.170)	3.740 (0.198)
Trust	4.100 (0.452)	4.160 (0.353)
Responsibility	4.255 (0.559)	4.350 (0.560)
Creativity	4.055 (0.134)	4.055 (0.163)
Initiative	3.200 (0.212)	3.385 (0.064)
Resilience	3.680 *	3.850 *
Social awareness	4.170 (0.297)	4.185 (0.163)
Belonging to well-informed social networks	4.410 (0.269)	4.465 (0.247)
Offering help and cooperation	4.380 *	4.410 *
Commitment and coherence	3.790 *	3.830 *
Coexistence and respect for public affairs.	4.100 *	4.200 *
Ability to identify opportunities	3.430 *	3.410 *
Ability to take risks	4.117 (0.370)	4.237 (0.345) **
Ability to create ideas.	3.525 (0.092)	3.625 (0.148)
Ability to learn and evolve.	2.755 (0.064)	2.930 (0.071) **
Ability to change	3.445 (0.361)	3.580 (0.438)

* It is not possible to calculate the standard deviation. ** Significant differences between pre-test and post-test measurements (*p* < 0.05).

**Table 4:** Tests of within-subject contrasts SE Pre-test/Post-test.

Source (Pretest/Post-Test)	SE Pretest/Post-Test	Type III Sums of Squares	df	Mean Square	*F*	Sig. (Bilateral)
SE	Linear	0.033	1	0.033	0.751	0.388
Age	Linear	0.260	1	0.260	5.986	0.016
Education access	Linear	0.211	1	0.211	4.845	0.030
Family education	Linear	0.000	1	0.000	0.003	0.957
Previous social participation	Linear	0.118	1	0.118	2.711	0.103
Employment status	Linear	0.148	1	0.148	3.403	0.069
Error	Linear	3.740	86	0.043		

Data obtained in the research (own elaboration).

**Table 5:** ANOVAs SE post-test depending on groups.

ANOVAs	Sums of Squares	df	Mean Square	*F*	Sig. (Bilateral)
	Age				
Between groups	0.110	2	0.055	0.286	0.752
Within groups	18.224	95	0.192		
Total	18.333	97			
	Education Access				
Between groups	0.174	3	0.058	0.299	0.826
Within groups	18.160	94	0.193		
Total	18.333	97			
	Family education				
Between groups	0.437	2	0.218	1.159	0.318
Within groups	17.897	95	0.188		
Total	18.333	97			
	Previous social participation				
Between groups	0.030	1	0.030	0.159	0.691
Within groups	18.303	96	0.191		
Total	18.333	97			
	Employment status				
Between groups	0.715	1	0.715	3.898	0.051
Within groups	17.618	96	0.184		
Total	18.333	97			

Data obtained in the research (own elaboration).

## Data Availability

The data presented and analysed in this study are available from the corresponding author upon reasonable request.
